# Negative to positive lymph node ratio is a superior predictor than traditional lymph node status in stage III colorectal cancer

**DOI:** 10.18632/oncotarget.10806

**Published:** 2016-07-24

**Authors:** Qingguo Li, Lei Liang, Huixun Jia, Xinxiang Li, Ye Xu, Ji Zhu, Sanjun Cai

**Affiliations:** ^1^ Department of Colorectal Surgery, Fudan University Shanghai Cancer Center, Shanghai, China; ^2^ Department of Oncology, Shanghai Medical College, Fudan University, Shanghai, China; ^3^ Center for Biomedical Statistics, Fudan University Shanghai Cancer Center, Shanghai, China; ^4^ Department of Radiation Oncology, Fudan University Shanghai Cancer Center, Shanghai, China

**Keywords:** colorectal cancer, surgical resection, prognosis analysis, lymph node ratio

## Abstract

Negative lymph node counts has recently attracted attention as a prognostic indicator in colorectal cancer (CRC). But little is known about prognostic significance of negative to positive lymph node ratio (NPR) in CRC. Our aim was to determine impact of NPR on oncological outcomes in patients with stage III CRC. This retrospective study included 2,256 patients with stage III CRC under curative resection at Fudan university Shanghai cancer center. Kaplan-Meier methods and multivariable Cox regression models were built for the analysis of survival outcomes and risk factors. Accuracy of the NPR was assessed with the Harrell's concordance-index(C-index).X-tile program identified 2.38 or 0.55/2.38 as the optimal cutoff value for NPR to divide the cohort into high/low risk or high/middle/low risk subsets in terms of CRC cause specific survival (CCSS). In a multivariate analysis, NPR was significant independent prognostic factors for CCSS (*P*<0.05), notably, N classification was not an independently prognostic factor (*P>0.05*). Further analysis found NPR could give detailed prognostic classification for both N1 and N2 stage (*P*<0.05). Interestingly, patients in N2+ NPR >2.38 stage have similar survival outcome with N1+ NPR >2.38 stage (χ2=0.030, *P*=0.863), and better than those at N1+ NPR ≤2.38 and N2+ NPR ≤2.38 stage (*P*<0.001). The TN_NPR_M stage was more accurate for predicting CCSS (C-index = 0.659) than current TNM stage system(C-index = 0.628) (*P*<0.001). Collectively, NPR was an independent prognostic factor for stage III CRC patients, it could provide more accurate prognostic information than the current node stage system.

## INTRODUCTION

Colorectal cancer (CRC) is one of the most common cancer with its incidence and mortality both ranked third among all malignancies worldwide [1]. In China, as the life style changed, the incidence rate of CRC has grown steadily. The rate of CRC increased as much as 4.2% per year from 1973 to 1993 in Shanghai, and it ranked as the second most common of cancer related deaths now [2, 3]. Surgical resection represents optimal approach for people with localized CRC, and to guarantee accurate tumor staging, a benchmark of at least 12 lymph nodes (LNs) retrieval has been recommended by the International Union Against Cancer and the American Joint Committee on Cancer (AJCC) since 2000. Theoretically, the survival of CRC patients is improved by removing more LNs. Previous researchers have indicated that the increased LNs retrieval is correlated with the reduced incidence of recurrence and tumor related death in patients with stage II CRC cancer [4–6]. However, debate exists regarding the clinical value of increased LN retrieval in stage III CRC. Le Voyer et al reported that there was an 23% increase in the 5-year overall survival if more than 40 LNs were retrieved rather than less than 10 LNs count for patients with N1 stage colon cancer,; and in patients with N2 stage, the 5-year overall survival rates following analysis of > 35 and < 35 LNs were 71% and 51%, respectively [7]. Vather et al showed that the LNs counts in stage III patients who died or were alive within 5 years was 13.1 *vs* 14.8, respectively, and this difference was statistically significant [8]. Chen et al. demonstrated that the median survival times for colon cancer patients with 1-7, 8-14 and ≥ 15 LN retrieval were 46, 52 and 67 months, respectively [9]. However, several studies have not demonstrated a similar correlation between LNs counts and survival in stage III CRC [5, 10–12]. The total number of LNs (TLN) retrieved comprises both positive and negative LNs (NLNs) in stage III patients, so the relationship between TLNs and prognosis is confounded by the prognostic effect of the number of positive LNs (PLNs). The concept of NLN counts has recently attracted attention as a prognostic indicator in various cancer. [13–16]. Our previous study also indicated that NLNs was an independent predictor in stage III rectal cancer [17]. It is reasonable to conjecture that negative to positive lymph node ratio (NPR) could be an important predictor in CRC. The purpose of present study was to investigate the prognostic value of NPR in patients with stage III CRC.

## RESULTS

### Identification of NPR cut-off points

Patients' clinicopathological parameters are demonstrated in Table 1. A total of 2,256 eligible patients were included in this study during the 10-year period, including 1,347 male and 929 female. The median age at diagnosis was 58 years old (Inter quartile range, IQR 49-68). There were 1,296 patients with N1 stage and 960 with N2 stage. The median number of LNs counts, positive LNs number, negative LNs, and NPR were 15.0 (IQR 12-19), 3 (IQR 2-6), 11(IQR 7-15), and 4.00 (IQR 1.50-9.00), respectively. Median follow-up time for present study cohort was 55 months. The 5-year CCSS was 69.0%.

**Table 1:** Clinicopathological characteristics and Kaplan-Meier CCSS analysis of colorectal cancer patients with lymph nodes involvement in Fudan University Shanghai Cancer Center

Characteristic	No.	5-year CCSS	Log-rank *χ*^2^	*P* value
Primary Site			0.317	0.574
colon	985	69.2%		
rectum	1271	67.9%		
Sex			1.128	0.288
male	1347	68.8%		
female	929	68.0%		
Age			7.827	0.005
≤60	1283	72.7%		
>60	973	63.9%		
Pathological grading			59.018	<0.001
Well/Moderate	1475	73.0%		
Poor/Anaplastic	686	58.4%		
Unknown	95	66.5%		
Histological Type			6.154	0.013
Adenocarcinoma	1876	69.0%		
Mucinous/Signet ring cell	380	66.1%		
T stage			47.049	<0.001
T1	26	89.1%		
T2	209	82.0%		
T3	351	85.2%		
T4	1670	63.5%		
N stage			51.193	<0.001
N1	1296	74.3%		
N2	960	60.3%		
Chemotherapy			26.049	<0.001
Yes	2138	70.3%		
No	118	46.8%		
No. of LNs dissected			14.820	<0.001
<12	469	60.9%		
≥12	1787	70.7%		
Negative/positive LNs ratio				
two groups			118.677	<0.001
≤2.38	821	54.4%		
>2.38	1435	76.8%		
three group			162.188	<0.001
≤0.55	246	40.0%		
0.55-2.38	575	60.3%		
>2.38	1435	76.8%		

**Figure 1:**
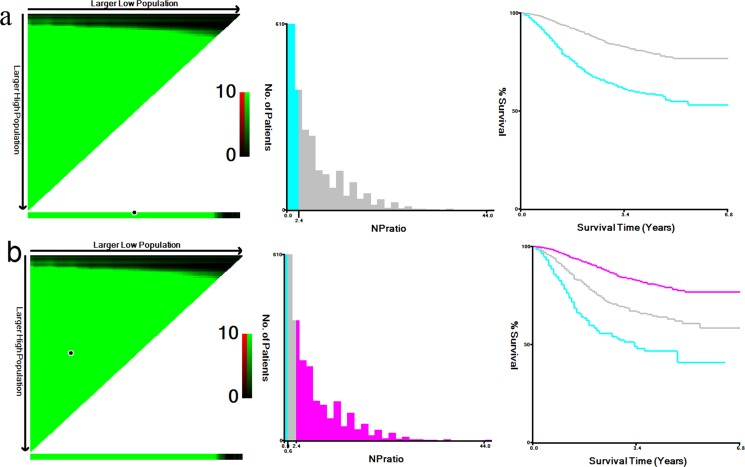
X-tile analysis of survival data from Fudan university Shanghai cancer center (FUSCC) X-tile analysis was done on patient data from FUSCC, equally divided into training and validation sets. X-tile plots of training sets are shown in the *left panels*, with plots of matched validation sets shown in the *smaller inset*. The optimal cut-point highlighted by the *black circle* in the *left panels* is shown on a histogram of the entire cohort (*middle panels*), and a Kaplan-Meier plot (*right panels*). *P* values were determined by using the cut-point defined in the training set and applying it to the validation set. *Figures* shows **a.** the maximum of χ2 log-rank values of 118.677(*p* < 0.001) was achieved when applying the number of 2.38 as the cutoff value for NPR to divide patients into high and low group;**b.** the maximum log-rank statistical value was 162.188 when the cutoff value were 0.55/2.38 (*p* < 0.001) for NPR. To divide patients into high, middle and low risk group.

X-tile program identified 2.38 as the optimal cutoff value for NPR with the maximum log-rank statistical value 118.677 to divide patients into high and low risk, and cutoff 0.55, 2.38 to divide patients into high, middle and low risk. (Figure 1).

### Prognostic value of the NPR

Since the N classification and NPR are both LN staging system, then we classified the patients into two risk subgroups according to NPR cutoff, N_NPR_1 (> 2.38), N_NPR_2 (≤ 2.38), or three risk subgroups N_NPR_1 (> 2.38), N_NPR_2 (0.55-2.38), N_NPR_3 (≤ 0.55), denoted as the N_NPR_ classification system, to distinguish from traditional N stage. Multivariate Cox regression analysis showed that age, tumor differentiation, T stage, and N_NPR_ stage had a significant correlation with CCSS *(P < 0.05)* (Table 2). A higher NPR (for two classifications, N_NPR_2, hazard ratio [HR] 0.428; 95% confidence interval [CI] 0.315-0.582, *P* < 0.001; for three classifications, N_NPR_2, HR 0.607,95%CI 0.465-0.793, N_NPR_3, HR 0.284,95%CI 0.195-0.415, *P* < 0.001, N_NPR_1 as reference) demonstrated a protective effect on survival. Notably, N classification was not an independently prognostic factor in multivariate Cox regression analysis (*P > 0.05*) (Table 2).

**Table 2:** Multivariate survival analyses for evaluating prognostic factors influencing colorectal cancer cause specific survival

Variable	HR(95%CI)	*P*	HR(95%CI)	*P*
Age		<0.001		<0.001
≤60	Reference		Reference	
>60	1.496(1.231-1.819)		1.526(1.255-1.856)	
Pathological grading		0.001		0.009
Well/Moderate	Reference		Reference	
Poor/Anaplastic	1.517(1.219-1.889)		1.423(1.137-1.781)	
Unknown	1.308(0.824-2.077)		1.268(0.799-2.011)	
Histological Type		0.709		0.489
Adenocarcinoma	Reference		Reference	
Mucinous/Signet ring cell	0.950(0.726-1.244)		0.909(0.693-1.191)	
T stage		<0.001		<0.001
T1	Reference		Reference	
T2	1.512(0.353-6.481)		1.492(0.348-6.394)	
T3	1.392(0.331-5.854)		1.404(0.334-5.902)	
T4a	3.155(0.782-12.734)		3.053(0.757-12.323)	
T4b	5.070(1.192-21.562)		4.851(1.141-20.630)	
N stage		0.872		0.516
N1	Reference		Reference	
N2	0.975(0.717-1.325)		0.900(0.656-1.235)	
No. of LNs dissected				
<12	Reference	0.009	Reference	0.016
≥12	0.730(0.577-0.923)		0.750(0.594-0.947)	
Adjuvant chemotherapy		<0.001		<0.001
Yes	Reference		Reference	
No	1.930(1.407-2.648)		1.937(1.411-2.659)	
NPR(two group)		<0.001		
N_NPR_1(>2.38)	Reference			
N_NPR_2(≤2.38)	2.335(1.717-3.176)			
NPR(three group)				<0.001
N_NPR_1(>2.38)			Reference	
N_NPR_2(0.55-2.38)			2.136(1.551-2.941)	
N_NPR_3(≤0.55)			3.517(2.407-5.138)	

a HR, hazard ratio; CI, confidence interval.

### Further analysis for the prognostic value of N_NPR_ stage according N classification

**Figure 2:**
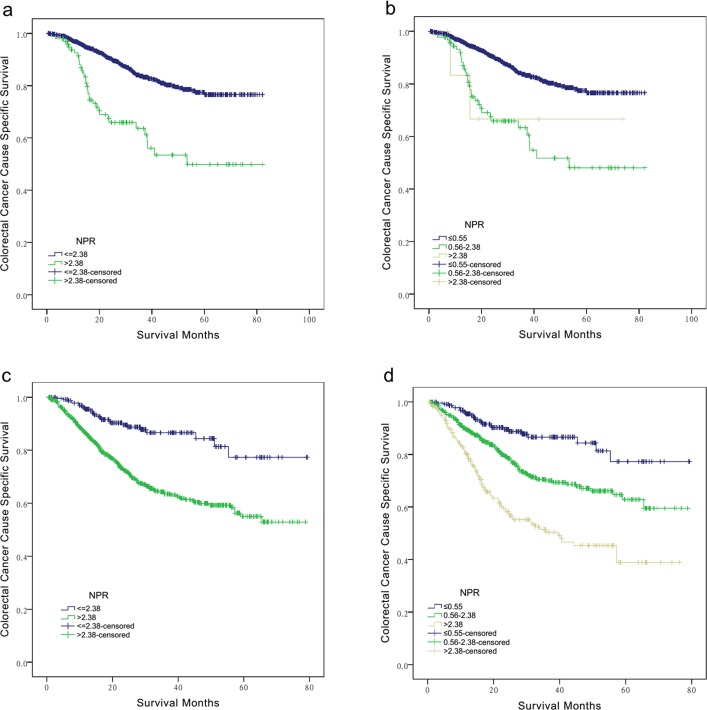
Subgroup analysis for evaluating the effect of N stage according N classification for colorectal cancer patients at III stage For patients at N1 stage, **a.** NPR > 2.38 *VS* ≤ 2.38, χ2 = 38.552, *P* < 0.001. **b.** NPR ≤ 0.55, 0.55-2.38 and > 2.38, χ2 = 38.879, *P* < 0.001. For patients at N2 stage, **c.** NPR > 2.38 *VS* ≤ 2.38, χ2 = 28.579, *P* < 0.001. **d.** NPR ≤ 0.55, 0.55-2.38 and > 2.38, χ2 = 62.401, *P* < 0.001.

We then made further subgroup analysis according to each N stage to determine the effect of N_NPR_ stage on CCSS. Specifically, for N1 patients, there was an absolute 26.7% increase in 5-year CCSS if NPR > 2.38 were analyzed than those patients of ≤ 2.38 (49.9% *VS* 76.6%, *P* < 0.001) (Table 3) (Figure 2). For N2 patients, the 5-year CCSS for CRC patients at N_NPR_1, N_NPR_2, N_NPR_3 stage were 38.9%,62.8% and 77.3%, respectively (*P* < 0.001), the 5-year CCSS was even two-folds in N_NPR_3 stage than that of N_NPR_1 stage(Table 3, Figure 2). In multivariate analysis, the N_NPR_ stage were all validated as independent prognostic factors in both N1 and N2 stage patients. (*P* < 0.05)(Table 3)

**Table 3:** Subgroup analysis for evaluating the effect of NPR on survival according to N stage

Variable	No.	5-year CCSS	HR	95%CI	*P*
**N1 stage**					
**N_NPR_(two group)**					<0.001
≤2.38	1198	76.6%	1.000	Reference	
>2.38	98	49.9%	0.406	0.252-0.654	
**N_NPR_(three group)**					0.001
>2.38	1198	76.6%	0.390	0.240-0.633	
0.55-2.38	91	48.1%	1.000	Reference	
≤0.55	7	66.7%	0.581	0.137-2.467	
**N2 stage**					
**N_NPR_(two group)**					<0.001
≤2.38	237	77.3%	1.000	Reference	
>2.38	723	55.0%	0.448	0.295-0.683	
**N_NPR_(three group)**					<0.001
>2.38	237	77.3%	0.529	0.343-0.817	
0.55-2.38	484	62.8%	1.000	Reference	
≤0.55	239	38.9%	1.744	1.308-2.323	

*P* values refer to comparison between each group to reference group and were adjusted for age, pathological grading, tumor histotype, T stage, No. of LNs dissected, adjuvant chemotherapy as covariates.

2. HR, hazard ratio; CI, confidence interval

### Combined analysis of N stage and N_NPR_ stage

In the above survival analyses, the patients in N2 stage with NPR > 2.38 exhibited a good 5-year CCSS than patients in N1 stage with NPR ≤ 2.38 (Table 3). Then we made combined analysis of N stage and N_NPR_ stage to divide the patients into four subgroups, N1+ N_NPR_1, N1+ N_NPR_2, N2+ N_NPR_1, and N2+ N_NPR_2.

**Figure 3:**
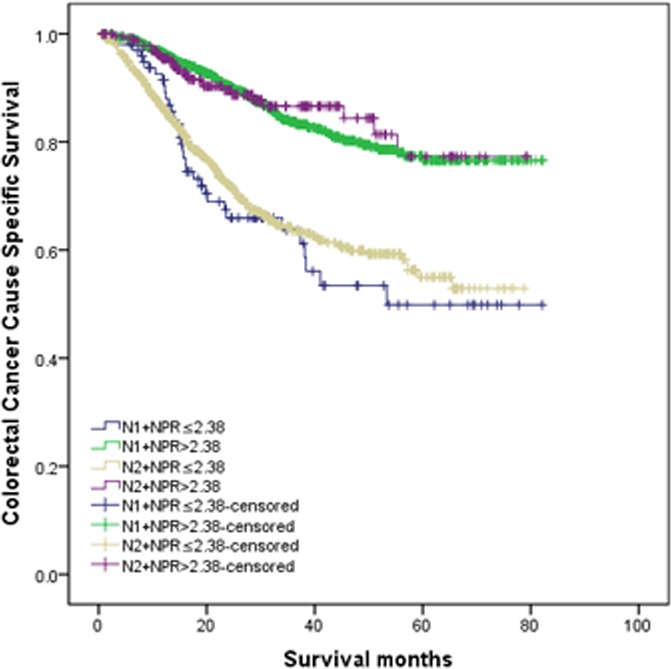
Combined analysis of N stage and N stage Patients in N2+ NPR > 2.38 stage have similar survival outcome with N1+ NPR > 2.38 stage (χ2 = 0.030, *P* = 0.863), and better than those at N1+ NPR ≤ 2.38 and N2+ NPR ≤ 2.38 stage (*p* < 0.001). The different between N1+ NPR ≤ 2.38 and N2+ NPR ≤ 2.38 stage was also not significance (χ2 = 0.290, *P* = 0.590).

Five-year CCSS were 49.9%, 76.6%, 55.0%, 77.3% for N1+ NPR ≤ 2.38, N1+ NPR > 2.38, N2+ NPR ≤ 2.38, N2+ NPR > 2.38 stage, respectively. Notably, patients in N2+ NPR > 2.38 stage have similar survival outcome with N1+ NPR > 2.38 stage (χ2 = 0.030, *P* = 0.863), and better than those at N1+ NPR ≤ 2.38 and N2+ NPR ≤ 2.38 stage (*P* < 0.001). The different between N1+ NPR ≤ 2.38 and N2+ NPR ≤ 2.38 stage was also not significance (χ2 = 0.290, *P* = 0.590) (Figure 3).

### Comparison of prognostic prediction accuracies between the TNM and TN_NPR_M stage classifications

We defined the TN_NPR_M system in reference with the current TNM stage, that T1-2N_NPR_1, T1N_NPR_2 were defined as IIIA, T3-4 N_NPR_1, T2-3 N_NPR_2, T1-2 N_NPR_3 were defined as IIIB, T4aN_NPR_2, T3-4aN_NPR_3, T4b N_NPR_1-3 were defined as IIIC. Table 4 presents classification for both the current TNM and TN_NPR_M stage systems. Statistical assessment of the prognostic performance of the stage systems by the c-index revealed a value of 0.659(95%CI:0.634-0.683) for TN_NPR_M, which was significantly better than 0.628(95%CI:0.603-0.652) for TNM.

**Table 4:** Cross-tabulation of the TNM and TN_NPR_M staging system

		TN_NPR_ M Stage			Total
		IIIA	IIIB	IIIC	
TNM stage	IIIA	162	10	0	172
IIIB		21	1148	65	1234
IIIC		0	194	656	850
Total		183	1352	721	

## DISCUSSION

Adequate LNs evaluation is required for accurate staging of CRC, and the number of LNs retrieval is a predictor in CRC after surgical resection. Lower LN evaluation is associated with worse survival outcome in terms of tumor recurrence and overall survival rate [4–6]. In stage III CRC, the total LNs are composed of both metastases LNs and negative LNs. The number of positive LNs is used as an important factor in current TNM stage and is associated with survival outcome for patients with CRC [18]. Our previous study also indicated that negative LNs was an independently prognosis factor in CRC. However, most of previous study did not consider both NLNs and PLNs simultaneously. More recently, some researchers have demonstrated the lymph node ratio (LNR) was a better indicator of prognosis rather than the number of metastatic LNs alone [19–21]. The assumption is that LNR accounts for both total lymph node retrieval, as well as the metastatic LNs number. However, higher weight is given to an LN metastasis when fewer overall LNs are retrieved [21].

Compared with the LNR, NPR is straight ratio between negative and positive LNs, which may serve as a better prognostic factor than PLNs, NLNs and LNR. In this study, NPR was validated as a risk factor for survival in CRC. In multivariate analysis, NPR remained an independent prognostic factor for CCSS, and the prognostic value of NPR remained significant in subgroup analysis of both N1 and N2 stage. Meanwhile, NPR also showed the greater log-rank χ2 value than current LN stage in stage III CRC. Importantly, C-index value is higher in TN_NPR_M stage than TNM stage, indicating that NPR had the greatest statistical significance for the prognosis of CRC. Precise tumor staging is one of most important predictor determining the patient's survival outcome. In TNM staging system, the N stage is the most important marker of the CRC patient's prognosis, so we suggest that using N_NPR_ stage instead of current N stage in stage III CRC, which could improve prognostic stratification.

NPR is more accurate because it takes into account both the PLNs and the NLNs, both of which has been validated to be important predictors, moreover, NPR is straight ratio between negative and positive LNs presents with several meanings. First, the number of metastasis LNs is directly associate with CRC patients' survival [18, 22]. Second, increasing NLNs retrieval can avoid stage-migration. The more LNs examined, the more likely that it reflects the true stage, and lower nodal counts may increase the risk of understaging. Third, NLNs is associate with the host immune response to cancer cells. The protective effect of NLNs may simply reflect a host lymphocytic reaction to the tumor, which is associated with LN count [23], and lymphocytic reaction to tumor cells has been correlated with prolong survival in CRC [24–26]. Fourth, the surgeon is a technician. It is possible that the patients who had higher number of LNs retrieved experienced more extensive excision of primary tumors and their draining nodes. Improved surgical techniques may also be the result of improved intraoperative staging [7] and to reduce the chances of iatrogenic spread of cancer cells. Then, the possibility of leaving tumor cells behind is low, which may have positively effect on survival. A high NLNs count may be an indicator of perfect surgical care or pathological examination. By increasing NLN counts, the chance of micrometastasis remaining within NLNs, which is a proven prognostic factor [27], may decrease.

Certainly, our study has several limitations. One of these is LN retrieval depends on multiple factors varying from surgeon's experience, techniques of LNs harvest individual surgeons, pathologists, and other factors, but we cannot adjust for these factors. However, this could be compensated for our center is one of the highest volume colorectal surgery units in China. All surgeons included in present study have received formal training in TME, and our Department of Pathology is the quality control center in Shanghai city. Every specimen was examined by two pathologists. Another is adjuvant therapy was only dichotomized as performed or not. The reagents and therapy cycles may affect survival as well.

In conclusion, our study shows that the NPR was an independent prognostic factor for stage III CRC patients, it could provide more accurate prognostic information than the current node stage system.

## MATERIALS AND METHODS

### Patients

The Fudan University Shanghai Cancer Center (FUSCC) CRC dataset was built prospectively and recorded the CRC patients treated at FUSCC, Shanghai, China since January, 2006. The records of patients with CRC who were treated at the FUSCC between January 2007 and December 2012 were retrospectively analyzed. Criteria for inclusion in the analysis were: (1) pathologically confirmed invasive CRC; (2) Received radical resection; (3) At least 1 LNs retrieval, and pathologic diagnosed as stage III patients; (4) CRC as a single primary tumor; and (5) age > 18 years old. Patients who received neoadjuvant therapy or died within 30 days after surgery were excluded from this study.

The research protocol was reviewed and approved by the Ethical Committee and Institutional Review Board of the FUSCC. All patients provided written consent for storage of their information in the hospital database, and for the research use of the information.

### Surgical management and follow up

All the patients underwent curative colorectal tumor resection plus lymphadenectomy. The standard surgical treatment for colon cancer is resection of the tumor and its mesentery with primary anastomosis. The precise extent of the resection depends on the location of the tumors and its arterial supply. The procedure of rectal cancer is performed as previously described [28]. All patients were asked to follow-up every 3-6 months at the Colorectal Cancer Center in the first 3 years after surgery by their operating surgical team, and every 6-12 months thereafter. Postoperative follow-up protocol included general physical examinations, digital rectal examination, and routine laboratory tests. Chest X-rays were performed every 6 months, and abdominal/pelvic CT and colonoscopy were performed every 6-12 months for the first 3 years. Surviving patients were followed-up between March and May 2015.

### Statistical analysis

Demographic and clinical variables including age, sex, tumor location, depth of tumor invasion, total number of LNs examined, number of involved LNs, grade, histotype, overall survival time, and cancer specific death were retrieved from FUSCC database. The TNM stages were restaged according to the 7th edition of AJCC/UICC staging system. The number of NLNs was obtained by subtracting the number of positive LNs from the total number of removed LNs. The NPR defined as the ratio of the number of NLNs to the number of positive LNs.

The NPR cutoff points were analyzed using the X-tile program (http://www.tissuearray.org/rimmlab/), which identified the cutoff with the minimum P values from log-rank χ2 statistics for the categorical NPR in terms of survival [29]. The relationship between various clinical and histological variables and survival was evaluated using the Kaplan-Meier method. Differences between survival curves were tested for statistical significance by using log rank test. The Cox proportional hazard regression model was used to identify the variables that could independently influence survival in CRC patients. The chi-square test was used for categorical variables. The 5-year colorectal cancer cause specific survival (CCSS) rate was estimated from Kaplan-Meier curves. Deaths attributed to the CRC of interest are treated as events and deaths from other causes are treated as censored observation.

The Harrell's concordance index(C-index) were used to compare the staging systems [30]. The C-index is a measure of discrimination used to evaluate whether a staging system can discriminate between two patients at different stages of disease. It is calculated as the probability that for a random pair of patients at different stages of disease, the patient at the lower stage has a longer observed survival. The range of the C-index is 0 to 1, 1 indicating a perfect discrimination, whereas 0.5 indicating no better concordance than chance, 0 indicating perfect discordance. The larger the C-index, the more accurate was the prognostic prediction [31]. Statistical analyses were performed with the statistical software package SPSS (Statistical Package for the Social Sciences) for Windows, version 17 (SPSS Inc, Chicago, IL, USA) and R (a language and environment for statistical computing) Version 3.0.2 for Mac (R Foundation for Statistical Computing, Vienna, Austria). Two-sided p values of less than 0.05 were considered to be statistically significant.
